# Kidney Transplant Recipient With Multiple Contemporaneous Malignancies Secondary to Muir-Torre Syndrome

**DOI:** 10.7759/cureus.16642

**Published:** 2021-07-26

**Authors:** Ahmed T Qudaih, Bayan H Al Ashour, Abdulrahman k Naim, Amani A Joudeh

**Affiliations:** 1 Medical Oncology, King Fahad Specialist Hospital, Dammam, SAU; 2 College of Medicine, Imam Abdulrahman Bin Faisal University, Tarout Island, SAU; 3 Nuclear Medicine, King Fahad Specialist Hospital, Dammam, SAU; 4 Department of Pathology, King Fahad Specialist Hospital, Dammam, SAU

**Keywords:** oncology, muir-torre syndrome, colorectal cancer, thyroid, lung metastasis, extraocular sebaceous carcinoma, kidney transplant.

## Abstract

Muir-Torre Syndrome (MTS) is a rare autosomal-dominant genetic condition linked to germline mutations in DNA mismatch repair (MMR) genes, resulting in microsatellite instability. It is considered a variant of Lynch syndrome characterized by the association of at least one sebaceous skin tumor and at least one internal malignancy. In addition, it has been shown that a latent phenotype of MTS might be unmasked in transplant organ recipients and immunosuppressed patients. The diagnosis and treatment of such cases require a multidisciplinary approach. Here, we present a case of a kidney transplant recipient who developed multiple sebaceous carcinomas 16 years after kidney transplantation and daily immunosuppressive medication. The patient then developed multiple contemporaneous internal malignancies in the esophagus and colon with metastases to the lung, thyroid, and lymph nodes, all of which were eventually linked to MTS. To our knowledge, this is the first reported case of MTS in the Arab world and the first reported case of esophageal cancer in relation to MTS in a transplant recipient. Because patients with MTS have a high tendency of developing malignancies, patients with a strong family history of malignancies, any known mutations, or an immunocompromised status should be included in an extensive screening program.

## Introduction

In 1967 and 1968, Muir and Torre respectively reported and described a rare autosomal dominant genetic condition characterized by a predisposition to cutaneous and visceral neoplasms called Muir-Torre Syndrome (MTS) [1-3]. MTS is considered a variant of Lynch syndrome (also known as hereditary nonpolyposis colorectal carcinoma syndrome), which was characterized by Lynch et al. in four families affected by colorectal cancer and sebaceous neoplasms in 1981 [4]. Both Lynch syndrome and MTS are caused by germline mutations in DNA mismatch repair (MMR) genes. A new subtype of MTS has been described which can occur without any family history of cancer and does not show any mutations in MMR genes [5]. All suspected cases need to be tested for MMR protein loss using immunohistochemistry (IHC) together with genomic sequencing to confirm the diagnosis. Here, we present a case of a kidney transplant recipient who developed multiple sebaceous carcinomas (SCs) 16 years after kidney transplantation and daily immunosuppressive medication which was then complicated by multiple internal malignancies. 

## Case presentation

**Figure 1:**
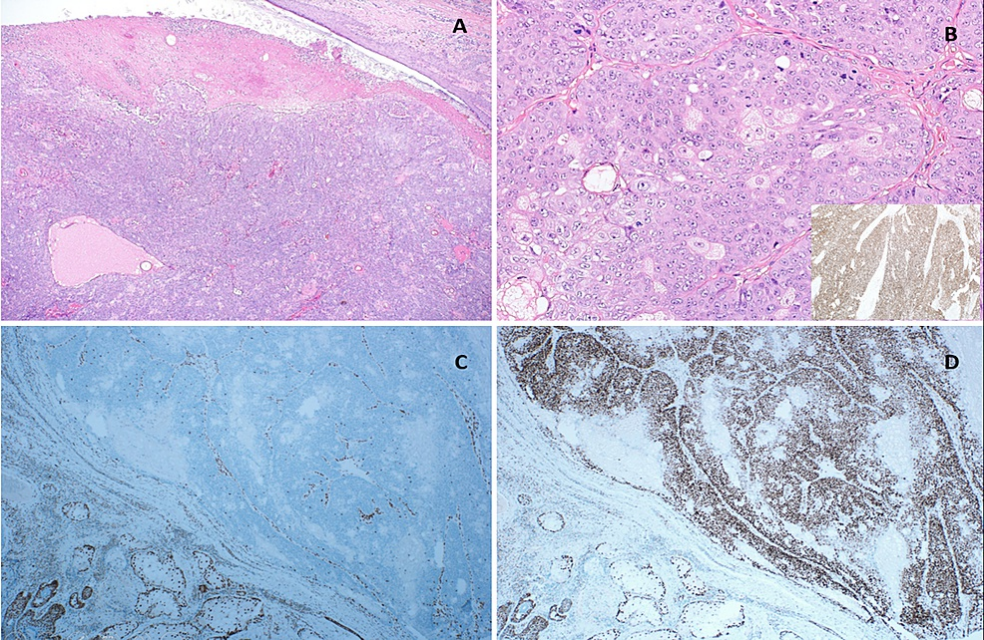
Microscopic​  examination of the shoulder and face lesions show similar histological changes. A) The tumors grew in a well-circumscribed lobular pattern. The surface over the tumor was ulcerated (hematoxylin and eosin stain (H&E), 40×).​ B) The neoplastic cells were predominantly basaloid with scattered groups of more mature vacuolated sebocytes.​ There was high-grade cytological atypia with numerous mitotic figures, many of which were atypical (​H&E, 100×); furthermore, the neoplastic cells were diffusely positive for androgen receptor (​lower right inset, IHC for AR). ​C) Immunohistochemical staining for MMR genes indicated a loss of nuclear staining for MSH2 and MSH6 with D) retained staining for PMS2 and MLH1. MMR: mismatch repair; MLH1: mutator L homolog 1; MSH: mutator S homolog; PMS: postmeiotic segregation increased 2; IHC: immunohistochemistry; H&E: hematoxylin and eosin stain; AR: androgen.

A 40-year-old man with end-stage renal disease successfully underwent kidney transplantation when he was 22 years old and has been on 5 mg of tacrolimus twice a day and 5 mg of prednisolone once daily. He underwent regular follow-up in another healthcare facility. The patient initially presented to our institution 16 years after transplantation with a fungating skin lesion which measured 5 × 5 cm on his left shoulder, and a biopsy was taken. The tumor was largely composed of basaloid sebaceous cells with significant squamous differentiation. He underwent wide local excision of the tumor. Pathology of the resected specimen confirmed the diagnosis of extraocular SC. Nine months later, he developed a facial lesion on his left cheek, for which he underwent wide local excision. The pathology results indicated extraocular SC composed of an admixture of basaloid and mature sebaceous cells that were largely circumscribed with focal necrosis. In addition, cystic changes were evident (Figure 1). Unfortunately, the patient was lost to follow-up until 16 months after the excision, when he presented to our medical oncology department with a history of dysphagia and weight loss of 15 kg over two months with no other associated symptoms. A detailed family history was negative for malignancy. His physical examination results were unremarkable. Tumor markers revealed elevated CA19-9 (9889 U/mL; normal <37 U/mL) and normal CEA (2.7 ng/mL; normal <5 ng/mL).

**Figure 2:**
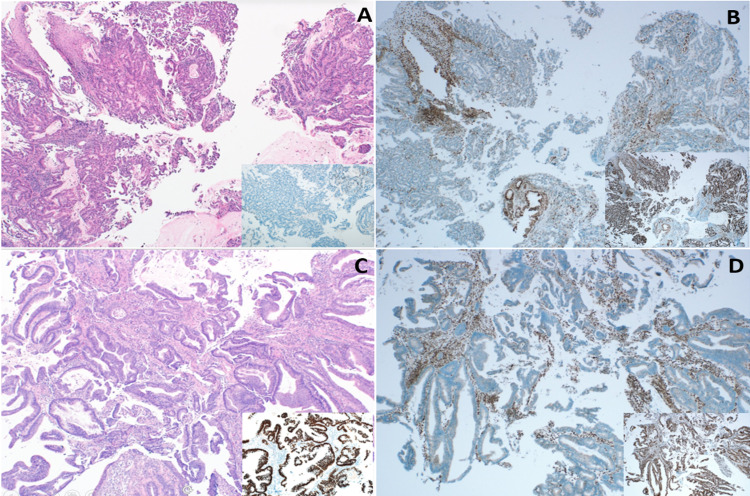
Microscopic examination of the esophageal and colonic mass A) Microscopic examination of the esophageal mass indicated infiltration by moderately differentiated adenocarcinoma (​H&E, 40×).​ The neoplastic cells of this mass were predominantly negative for CDX2 (Lower right inset, IHC for CDX2).​ Note the residual normal esophageal mucosa at the upper left corner of panel A. B) Immunohistochemical staining for MMR genes indicated a loss of nuclear staining for MSH2 and MSH6 with retained staining for PMS2 and MLH1 (Lower right inset). C) Microscopic examination of the colonic mass showed infiltration by well-differentiated adenocarcinoma (H&E, 100×).​ The neoplastic cells were diffusely and strongly positive for CDX2 (Lower right inset, IHC for CDX2). D) Immunohistochemical staining for MMR genes indicated a loss of nuclear staining for MSH2 and MSH6 with retained staining for PMS2 and MLH1 (Lower right inset). MMR: mismatch repair; MLH1: mutator L homolog 1; MSH: mutator S homolog; PMS: postmeiotic segregation increased 2; IHC: immunohistochemistry; H&E: hematoxylin and eosin stain.

**Figure 3:**
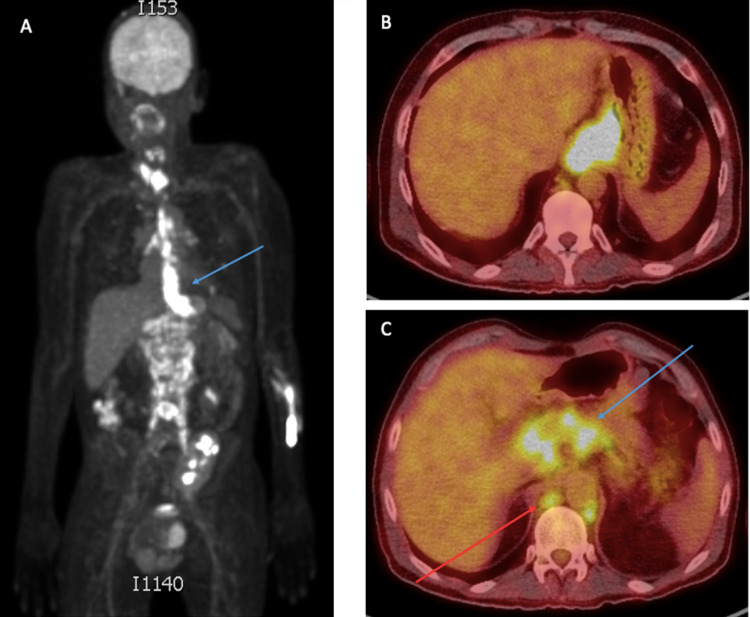
PET/CT whole body scan A) Coronal maximum intensity projection image of a whole-body 18F-FDG PET scan showing marked FDG-avid circumferential wall thickening at the mid and distal thirds of the esophagus extending to the gastric fundus corresponding to the known malignancy (blue arrow). B) Axial-fused 18F-FDG PET/CT image showing a marked FDG-avid lower esophageal mass corresponding to the known malignancy. C) Axial-fused 18F-FDG PET/CT image showing marked FDG-avid conglomerate gastrohepatic/celiac (blue arrow) and retrocrural (red arrow) lymphadenopathies consistent with metastasis.

**Figure 4:**
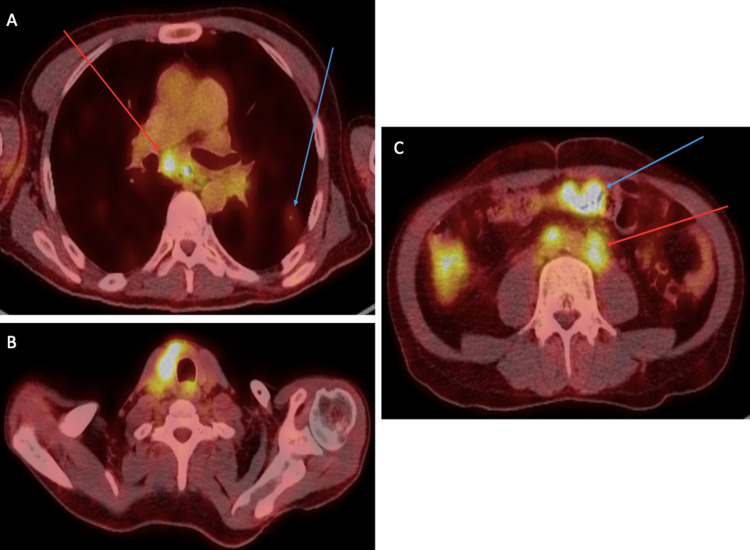
PET/CT whole body scan A) Axial-fused 18F-FDG PET/CT image showing an FDG-avid pulmonary metastasis (blue arrow) and an FDG-avid metastatic subcarinal lymph node (red arrow). B) Axial-fused 18F-FDG PET/CT image at the level of the thyroid gland showing a marked FDG-avid mass occupying the left thyroid lobe extending to the isthmus, which was likely malignant. C) Axial-fused 18F-FDG PET/CT image of the abdomen showing marked FDG-avid short segment circumferential wall thickening at the mid transverse colon (blue arrow) that was likely malignant. FDG-avid metastatic retroperitoneal lymphadenopathy was also noted (red arrow).​

Esophagogastroduodenoscopy revealed a friable mass extending 23 cm from the incisors to the gastroesophageal junction, which also caused a stenosis at the level of the gastroesophageal junction. Biopsies taken from the lesion suggested moderately differentiated adenocarcinoma, and immunohistochemistry indicated that the tumor was negative for homeobox protein-2 (CDX2). Immunohistochemical staining for MMR genes revealed loss of mutator S homolog (MSH)2/MSH6 protein expression (Figure 2A, 2B). PET/CT revealed marked FDG-avid circumferential wall thickening almost the entire length of the esophagus (Figure 3A, 3B) and pulmonary metastasis with marked FDG-avid supra- and infra-diaphragmatic lymph nodes consistent with metastasis (Figure 3C, Figure 4A). In addition, there was a marked FDG-avid right thyroid nodule (Figure 4B) and focally increased FDG activity in the mid-transverse colon (Figure 4C). Colonoscopy showed a large friable mass occupying half of the lumen extending between two folds at the proximal transverse colon and hepatic flexure. Analysis of the biopsy of the colonic mass indicated well-differentiated adenocarcinoma and immunohistochemistry indicated that the tumor was strongly positive for CDX2 (Figure 2C). Further immunohistochemical staining on colon tumor cells revealed loss of MSH2/MSH6 protein expression (Figure 2D), indicating defective DNA mismatch repair, and the tumor was presumed to be MSI-high. Next-generation sequencing of genomic DNA confirmed the presence of a heterozygous pathogenic c.(366+1_367-1)_(1276+1_1277-1) deletion variant in MSH2, which confirmed the genetic etiology of the clinical presentation of our patient. It also confirmed the diagnosis of MTS with an autosomal dominant pattern of inheritance. He was referred for genetic counseling and screening of his first-degree relatives. Tacrolimus was replaced with 4 mg of sirolimus once daily after discussing the case with the transplant team in an effort to prevent further tumor development and progression. The case was discussed in a multidisciplinary meeting, which recommended an esophageal stent be given to the patient. After stent placement, the decision was made to start the patient on palliative FOLFOX chemotherapy (oxaliplatin 85 mg/m2, leucovorin 400 mg/m2, 5-fluorouracil 400 mg/m2 bolus and 2400 mg/m2 IV infusion over 46 hours) to target both the esophageal and colonic tumors. He received 12 cycles of FOLFOX and good clinical and radiological responses were reported, as well as normalization of the patient’s CA19-9 level.

## Discussion

MTS is a rare autosomal dominant genetic condition characterized by at least one sebaceous skin tumor and at least one internal malignancy. It is considered a variant of Lynch syndrome [4]. Most of the cases that have been reported described Caucasian patients from developed countries. There are limited data about the prevalence of such cases in Asian and African populations. The disease occurs in both sexes, with a male-to-female ratio of 3 to 2. The age at the onset of malignancy ranges from 23 to 89 years, with a median age of 53 years [5]. MTS is linked to germline mutations in DNA mismatch repair (MMR) genes, including mutator L homolog 1, MSH2 and MSH6, and postmeiotic segregation increased 2. MSH2 is the most commonly mutated gene in patients with MTS [6-8]. There are two types of MTS: MTS1, which is a variant of Lynch syndrome and accounts for 65% of MTS cases, and MTS2, which accounts for 35% of MTS cases. The pathogenesis of MTS2 is undefined; genetic testing does not show any mutations in MMR genes, and tumor cells do not display microsatellite instability [6,9]. Additionally, MTS2 is characterized by an autosomal-recessive inheritance, likely related to genes that are not implicated in MMR, and patients with MTS2 tend to develop tumors later than patients with MTS1 [5]. Interestingly, several reports have shown that MTS could be unmasked in transplant recipients and immunosuppressed patients [6]. 

Basal cell carcinomas with sebaceous differentiation, sebaceomas, sebaceous adenomas, SCs, and keratoacanthomas have all been described in patients with MTS [10], and the internal malignancies frequently include but are not limited to genitourinary cancers, endometrial cancers, breast cancers, and upper gastrointestinal malignancies. Colorectal cancer is the most common internal malignancy; it occurs in half of patients with MTS [10,11]. Almost 50% of patients with MTS develop at least two visceral malignancies [5]. Approximately 56% of patients develop internal malignancies before developing cutaneous lesions, 6% have synchronous cutaneous and internal malignancies, and 22% present first with a cutaneous lesion [12,13]. Hence, the presence of these cutaneous lesions should prompt further investigation to identify occult internal malignancies with the aim of early treatment. 

A multidisciplinary team program is the best approach for the diagnosis and treatment of all suspected cases of MTS. Patients are suspected to have MTS if they have one or more of the following clinical criteria: a history of at least one sebaceous tumor, an age younger than 60 years at the time of diagnosis of the first sebaceous tumor, and personal and/or family history of Lynch-related cancers [14]. All suspected cases should be tested for MMR protein loss using immunohistochemistry (IHC). Although the loss of MMR protein expression is highly suggestive of germline mutations, it may also represent sporadic mutations in MMR genes due to acquired somatic mutations, although this is more common in patients who have undergone transplantation. Therefore, identification of germline mutations in MMR genes by genomic sequencing is needed to confirm an MTS diagnosis [5]. Because of the strong likelihood of developing malignancies among patients with MTS, patients with a strong family history of malignancies, known mutations in MMR genes, or an immunocompromised state should undergo an extensive screening program to detect any early malignancy before it reaches an untreatable or poor prognostic stage. The screening program conceivably includes cystoscopy, abdominal and pelvic CT scans, regular dermatological examination and follow-up, colonoscopy, mammography, endometrial biopsy, and upper gastrointestinal endoscopy [5,10]. Each screening test should be performed within a specific time frame and age according to the latest guidelines, patient health condition, and accessibility.

Only a limited number of MTS cases have been reported in relation to kidney transplants. Nakada and colleagues reported the last similar case involving a 43-year-old post-kidney transplant woman diagnosed with MTS after being admitted to the hospital with SC concurrent with colon cancer. This patient developed cancers 10 years after transplantation, during which time she received immunosuppressive medication consisting of tacrolimus, methylprednisolone, and mycophenolate mofetil. These authors concluded that immunosuppressant therapy could play a role in the development of malignancies in MTS patients [15]. The development of neoplasms because of immunosuppressive agents should not be underestimated. Tacrolimus and cyclosporine, both calcineurin inhibitors, have been reported to cause tumor progression, and switching patients to different drugs, such as mammalian target of rapamycin inhibitors, decreases tumor formation [15,16]. 

## Conclusions

MTS is a rare condition, but physicians should maintain a high index of suspicion for it, especially among transplant recipients receiving immunosuppressant therapy with any history of sebaceous neoplasms, even in the absence of a family history of malignancies. IHC testing for the loss of MMR protein expression together with germline mutation testing should be considered in such cases, and patients should undergo a regular multidisciplinary screening program to detect internal malignancies at an early stage in an effort to reduce morbidity and mortality. We believe that our case provides further evidence that immunosuppressant therapy could unmask latent MTS in transplant recipients and promote tumor progression, as reported previously.
